# Analysis of Variations in the Flavonoid Profiles of *Cuscuta campestris* and *Cuscuta epithymum* in Bulgaria as a Potential Chemotaxonomical Marker

**DOI:** 10.3390/plants14081220

**Published:** 2025-04-16

**Authors:** Bilyana Chakarova, Lyuben Zagorchev, Kalina Pachedjieva, Anita Tosheva, Tzvetelina Zagorcheva, Krasimir Rusanov, Denitsa Teofanova

**Affiliations:** 1 Faculty of Biology, Sofia University “St. Kliment Ohridski”, 8 Dragan Tsankov Blvd., 1164 Sofia, Bulgaria; bilyana.chakarova1@gmail.com (B.C.); kalina_p@biofac.uni-sofia.bg (K.P.);; 2 AgroBioInstitute, Agricultural Academy, 8 Dragan Tsankov Blvd., 1164 Sofia, Bulgaria; 3 Research and Development and Innovation Consortium, Sofia Tech Park JSC, 111, Tsarigradsko Shose Blvd., 1784 Sofia, Bulgaria; 4 Centre of Competence “Sustainable Utilization of Bio-Resources and Waste of Medicinal and Aromatic Plants for Innovative Bioactive Products” (BIORESOURCES BG), 1000 Sofia, Bulgaria

**Keywords:** bioactive compounds, chemotaxonomical markers, dodders, flavonoids, parasitic plants, polyphenolics

## Abstract

Holoparasitic plants of the genus *Cuscuta* are generally considered prominent agricultural pests. In addition to their negative economic effect on agriculture and their impact on natural plant societies, they have also been long known in East Asian as medicinal plants with beneficial properties. This underlines the fact that *Cuscuta* spp. are particularly rich in specialized metabolites, flavonoids, alkaloids, and cumarines, among others. In addition to several well-characterized species, most of the species variety within the genus remains largely unstudied. In the present study we aimed to compare the flavonoid profiles of natural populations of two of the most abundant *Cuscuta* species in Bulgaria—the native *C. epithymum*, and the naturalized *C. campestris*. Based on HPLC-MS/MS analysis, a total of 13 polyphenolics compounds were annotated, with hyperoside and isoquercitrin being some of the most abundant. Some notable differences were found, like the complete absence of dicaffeoylquinic acid in *C. campestris*, and kaempferol-3,7-O-diglucoside and kaempferol 3-O-β-(6′′-O-trans-p-coumaroyl)-glucopyranoside in *C. epithymum*. The population of the two species clustered separately from each other, with some variations, but with no clear pattern of dependence on the locality or host species. Based on the results it can be concluded that flavonoids may be used as chemotaxonomical markers within the genus, showing that even in different climatic conditions and different host ranges, the two studied species clearly differed from each other. Also, their rich content emphasizes the potential of these parasites as a source of bioactive compounds.

## 1. Introduction

*Cuscuta* is the single parasitic genus in the morning glory (Convolvulaceae) family, previously separated in its own family Cuscutaceae [1], and including around 200 species of worldwide-distributed stem holoparasitic plants, called dodders [2]. The greatest diversity has been found in North and South America, and around 50 species have been established in the USA alone [3]. Several species are also found in Europe, either native or introduced, of which nine are naturally distributed in Bulgaria [4,5]. Among the most widely distributed are *C. europaea* L. and *C. epithymum* L., which are considered native, as well as the introduced *C. campestris* Yunk. The latter is also considered one of the worst agricultural pests in the genus [6], although *C. epithymum* [7] was also reported as a weed.

In addition to their negative impact on crop plants, dodders were also found to contain a great variety of specialized metabolites, including flavonoids, alkaloids, glycosides, and lignins, as well as essential oils [8]. Flavonoids belong to the broader group of polyphenolic compounds, to which phenolic acids, cumarines, tannins, and lignins also belong [9]. The polyphenolic profile was proposed as a way to distinguish between different *Cuscuta* species, as the levels of different compounds within this group vary significantly between species [1,10]. However, the content of specialized metabolites in this parasitic genus may also vary depending on the host plant. For example it was found that the distribution of alkaloids in *Cuscuta palaestina* Boiss. is often similar to that of the host plant, suggesting extensive transfer between host and parasite [11]. Similar results were demonstrated in *C. reflexa* Roxb. and *C. campestris* [12,13].

Some of the most extensively studied *Cuscuta* species, in terms of specialized metabolite content, are *C. reflexa* and *C. chinensis* Lam., which are especially rich in flavonoids. While in traditional medicine, mainly seeds are used [8], it is noteworthy that the vegetative parts (e.g., the stem) are equally rich in the same compounds [14]. Common flavonoids for the entire genus include quercetin, hyperoside, and kaempferol, and their glycosides, kaempferol-3-O-glucoside, and quercetin-3-O-glucoside, as well as chlorogenic acid. In addition, numerous unique compounds were found separately in *C. reflexa* or *C. chinensis* [8]. This appearance of unique flavonoids in particular *Cuscuta* species determines their potential suitability as chemotaxonomic markers. For example, *C. chinensis* and *C. australis* R. Br. are two species in this parasitic genus that are very similar in appearance, and have overlapping distribution ranges, but kaempferol and astragalin in *C. australis* were remarkably higher than in *C. chinensis* [10]. Such differences may be essential for the differences in the medicinal properties.

Because of its high content of bioactive compounds, *Cuscuta* spp. are also considered to be plants with medicinal potential. *Cuscuta chinensis* and *C. australis* are widely employed in Chinese traditional medicine, mostly owing to their polyphenolic content [10]. In addition to these two species, at least 15 other species are considered medicinally significant, including *C. campestris* and *C. epithymum*, which are also found in Bulgaria [4,5]. As medicinal plants, some authors from Bulgaria [15] mention *Cuscuta epithymum, C. europaea,* and *Cuscuta monogyna* Vahl., and the species *C. europaea* and *C. epilinum* are included in the Medicinal Plants Act (2000). Extracts from *Cuscuta* spp. are also used in Bulgarian traditional medicine [16], but still, the distribution of this parasitic genus, its genetic diversity, and its polyphenolic content are largely unknown in the country. In the present study, we aimed to characterize the variations in flavonoid content in different localities of two species—*C. campestris* and *C. epithymum*—with respect to their chemotaxonomical value and medicinal potential. The two species clearly differed in their flavonoid profiles, and despite the differences in their geographical distribution and host range, clustered together, emphasizing the chemotaxonomical applicability of polyphenolics within the genus.

## 2. Results

### 2.1. Total Polyphenolics

Our first goal was to determine the polyphenolic content in different samples (Table 1).

Although largely non-polar, the recovery rate and stability of particular flavonoid compounds depend significantly on the solvent identity and solvent concentration [17]. To test this, we performed extractions with three different concentrations of both ethanol and methanol on two randomly chosen samples from both species and measured total polyphenolics in the extracts (Table 2). Depending on the solvent, the recovery of total polyphenolics in *C. campestris* ranged from 3 to 8 mg g^−1^ fresh weight and that of *C. epithymum* ranged from 4 to 10 mg g^−1^ fresh weight. While in *C. campestris* the recovery was higher with a lower concentration of the alcoholic solvents, in *C. epithymum* the opposite trend was observed—the highest recovery of polyphenolics was achieved with concentrated ethanol and methanol.

**Figure 1:**
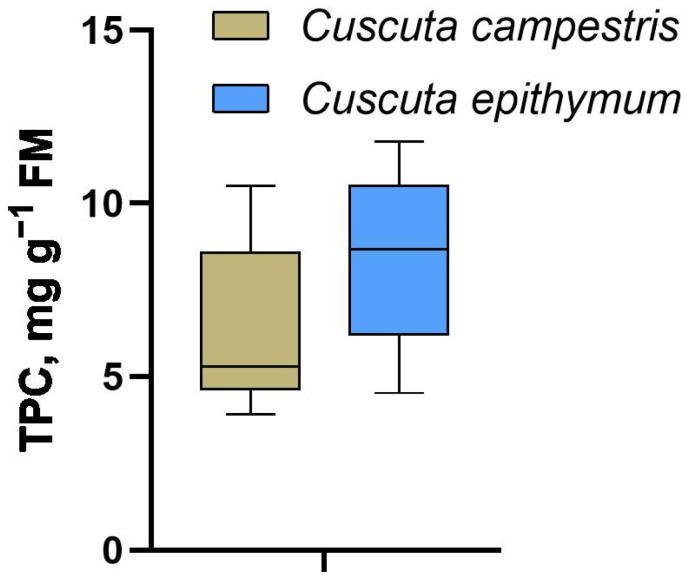
Box plot diagram of mean total polyphenolic content in *Cuscuta campestris* and *Cuscuta epithymum*, represented as gallic acid equivalents in mg g^−1^ fresh weight.

Based on the results, extraction with 100% methanol, giving high total polyphenolic content in both species, was employed to further extract flavonoids from all samples (Table 3). The total polyphenolic content (TPC) was highly variable between different samples of the same species, starting from 4 mg g^−1^ fresh weight and reaching 10 mg g^−1^ fresh weight in *C. campestris* to 12 mg g^−1^ fresh weight in *C. epithymum*. The standard deviation within the three samplings from the same population was also high. The overall TPC was higher in *C. epithymum*, compared with *C. campestris* (Figure 1) and in this species the variations were also more equally distributed, while in *C. campestris* most of the samples had lower TPC with only three exceptions—C4, C10, and C21.

### 2.2. HPLC Analysis of Flavonoids

**Figure 2:**
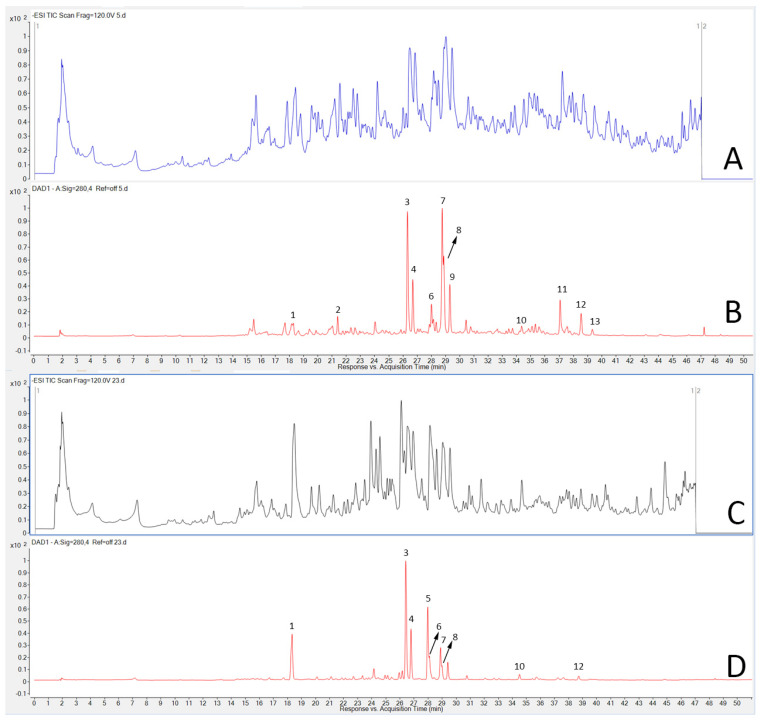
Representative chromatograms of *Cuscuta campestris* (C5; (**A**,**B**)) and *Cuscuta epithymum* (C23; (**C**,**D**)). The numbers on the DAD chromatograms (**B**,**D**) correspond to the designation of individual compounds in Table 1. The corresponding MS chromatograms (**A**,**C**) are also shown.

A total of 13 compounds were annotated using HPLC-DAD-MS separation/detection (Table 4; Figure 2), of which 11 were flavonoids and 2 were derivatives of chininic acid. Of these, two were missing in *C. epithymum* and five were missing in *C. campestris*.

All ten flavonoids (**2**, **3**, **4**, **6**, **7**, **8**, **9**, **10**, **11**, **12**, and **13**) are representatives of the flavonol class and fragmentation of these compounds (MS/MS) resulted in fragmented ions of the aglycons: quercetin at *m*/*z* 301, isorhamnetin at *m*/*z* 315, and kaempferol at *m*/*z* 285. Accordingly, compound **10** was characterized by UV absorption at 255 nm and 370 nm and a deprotonated ion at *m*/*z* 301, which suggested it corresponded to quercetin [10]. Compounds **3** and **4** showed identical [M-H]^−^ ions with *m*/*z* 463, and similar UV absorption with maximums at 255 nm and 355 nm. Both compounds gave deprotonated ions with *m*/*z* 300, *m*/*z* 271, and *m*/*z* 179 after fragmentation (Table 2). Ions with *m*/*z* 300 indicated a hexose unit ([(M-H)-162-H]^−^), while fragmented ions with *m*/*z* 271 and *m*/*z* 179 are characteristic of the aglycon quercetin [18]. Based on literature data [19], MS and MS/MS spectra, and UV absorption, compounds **3** and **4** were annotated as quercetin-3-O-galactoside (hyperoside) and quercetin-3-O-glucoside (isoquercitrin). Compound **12** was characterized by deprotonated ion at *m*/*z* 285 and UV absorption maximum at 265 nm and 366 nm, and was thus annotated as kaempferol [20]. Compounds **6** and **7** shared similarities in the pseudomolecular ion at *m*/*z* 447 and acquired ions after fragmentation. Based on UV absorption at 265 nm, characteristic for aglycone kaempferol fragmented ions (*m*/*z* 255 and *m*/*z* 227) and fragmented ions at *m*/*z* 284, indicating loss of hexose moiety ([(M-H)-162-H]^−^), these compounds were annotated as kaempferol-3-O-galactoside (compound **6**) and kaempferol-3-O-glucoside (astragalin; compound **7**) [21,22]. Compound **2** showed deprotonated ions at *m*/*z* 609 and fragmented ions at *m*/*z* 447, 285, 489, and 327. The UV absorption at 265 nm and 345 nm suggested a glycoside of kaempferol. The characteristic fragmented ion decay of this compound at *m*/*z* 285 indicated the loss of a dihexose (324 Da) and based on the spectra obtained and literature data for *Cuscuta*, this compound was annotated as kaempferol-3,7-O-diglucoside [10,18]. In addition, the higher polarity due to the presence of two hexose groups, explained the lower retention time (tR) and, therefore, the earlier elution. Compound **13** contained the [M-H]^−^ ion at *m*/*z* 315 and was characterized by UV absorption at 256 nm and 368 nm. Loss of a methyl group upon fragmentation resulted in the resulting fragmented [(M-H)-15]^−^ ion at *m*/*z* 300, suggesting this compound to be isorhamnetin, according to literature data [10]. Compounds **8** and **9** were characterized by an [M-H]^−^ ion at *m*/*z* 477 and similar fragmented deprotonated ions. Small differences were observed in the UV spectra, where, in addition to the shared absorption at 255 nm, compound **8** was also characterized by absorption at 353 nm, while in compound **9**, absorption occurred at at 345 nm. Due to the resulting fragmented ions, which are characteristic of the aglycone isorhamnetin, and the the fact that the difference of 162 Da corresponded to the loss of hexose, compounds **8** and **9** were suggested to be isorhamnetin-7-glucoside and isorhamnetin-3-O-glucoside [21]. The MS and MS2 data for compound **11**, with a pseudomolecular ion of 593.1281 and a characteristic MS2 fragment of 285.0389 as well as a UV absorbtion spectra with the UV max of band I at 315 nm and band II at 258 nm suggested that this compound was kaempferol 3-O-β-(6′′-O-trans-p-coumaroyl)-glucopyranoside, which had been previously annotated in *Cuscuta* extracts [23].

Compounds **1** and **5** differed dramatically in UV spectra from the remaining eleven compounds and were characterized by absorption at 218 nm, with shoulders at 300 nm and 327 nm. The presence of the highest absorption at 327 nm and a shoulder around 300 nm is a distinguishing feature of organic acid derivative compounds and, therefore, can be used to identify this class of compounds. In addition, in MS2 analysis of such compounds, due to the loss of caffeic acid, a fragmented [M-H]^−^ ion is often observed at *m*/*z* 161 [24]. For compound 1, an [M-H]^−^ ion was observed at *m*/*z* 315 and fragmented ions at *m*/*z* 191 and *m*/*z* 161, which is common in ESI-MS of caffeic acid derivatives substituted with caffeoyl groups [25]. Compound **5** was characterized by an [M-H]^−^ ion at *m*/*z* 515 and, after fragmentation, the resulting ions were at *m*/*z* 353, *m*/*z* 191 and *m*/*z* 135. Therefore, based on the UV absorption spectrum, MS data, and literature search [10,25], compounds **1** and **5** were assumed to be, respectively, 5-caffeoylquinic acid and dicaffeoylquinic acid.

A comparative analysis of the flavonoid profiles was performed between the two species based on the areas of the integrated peaks of the compounds from UV chromatograms at 280 nm. The peak areas from the 12 samples for each species were averaged and then compared between the two *Cuscuta* species.

**Figure 3:**
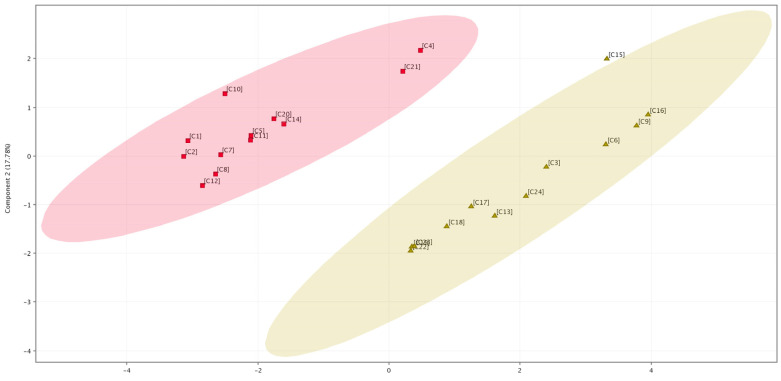
Principal component analysis (PCA) based on 8 compounds. Twenty-four samples are plotted on the two most significant components (Component 1 on the x-axis and Component 2 on the y-axis). Samples from *C. campestris* are shown in red, while samples from *C. epithymum* are shown in brown. The ellipses flanking the samples of both species represent 95% confidence ellipses.

**Figure 4:**
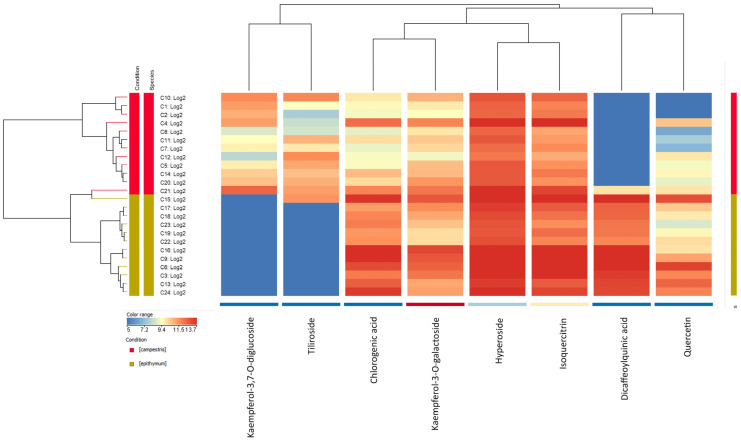
Hierarchical clustering by *Cuscuta* samples and the 8 flavonoid compounds. The dendrogram on the left shows the division of the samples into two main groups (sample C21 excluded), coincident with the *Cuscuta* species (*C. epithymum* and *C. campestris*). The dendrogram at the top shows the proximity of the base contents in the samples of the compounds annotated by Agilent HPLC, which varied statistically significantly between species.

Analysis using the *t*-test showed that 8 of the 13 annotated compounds varied statistically significantly (*p* < 0.05) between the two species of *C. campestris* and *C. epithymum*, including kaempferol 3-O-β-(6′′-O-trans-p-coumaroyl)-glucopyranoside, chlorogenic acid, hyperoside, isoquercitrin, kaempferol-3-O-glalactoside, quercetin, kaempferol-3,7-O-diglucoside, and dicaffeoylquinic acid. The set of eight compounds was used for principal component analysis (PCA) as well as analysis based on hierarchical clustering (Figure 3 and Figure 4). PCA analysis showed the formation of two distinct groups of samples according to their species affiliation. The two components of the PCA plot together described 88.18% of the total variation in the system (Figure 3). From the PCA analysis, it is noticeable that the samples from *C. epithymum* had a higher value of Component 1, while those from *C. campestris* had a lower value of Component 1 and a higher value of Component 2. Samples C4 and C21 from the *C. campestris* group were more distant on the basis of flavonoid content compared with the other samples from this group, which were closely spaced on the PCA plot and, therefore, were characterized by a more similar flavonoid profile. Concerning the *C. epithymum* group, plants C6, C9, and C16 were closely spaced to each other but were more distant from the other eight samples of the species and, therefore, differences in the flavonoid profile were observed. Sample C15 was an outlier in the *C. epithymum* group because it lay outside the 95% confidence interval indicated by the brown ellipse in Figure 4. No grouping of samples by host was observed from the results. For example, C1 and C20, as well as C2 and C7 were not clustered together. Contributing to this may be also the fact, that in addition to the main parasitized species, the sampled *Cuscuta* specimens were also infesting nearby plants from other species, making it difficult to isolate the host influence.

The cluster analysis also showed a separation based on sample affiliation to the two studied species (Figure 4). The PCA observations were confirmed where sample C15 was the most divergent from *C. epithymum* and more similar to sample C21 from *C. campestris* and these two samples formed a dendogram group that was part of *C. epithymum* (Figure 4). Fold change in the content of the eight compounds was also analyzed, and varied statistically significantly between the two species studied. Of these, kaempferol-3,7-O-diglucoside and kaempferol 3-O-β-(6′′-O-trans-p-coumaroyl)-glucopyranoside were absent in the *C. epithymum* samples, except for sample C15, which contained kaempferol 3-O-β-(6′′-O-trans-p-coumaroyl)-glucopyranoside. Meanwhile, dicaffeoylquinic acid was absent in the *C. campestris* samples except for sample C21. Quercetin was, on average, more than 20 times more abundant in the samples from *C. epithymum*. The remaining compounds, chlorogenic acid, hyperoside, isoquercitrin, and kaempferol-3-O-galactoside were also higher in average content in *C. epithymum* with 6.53, 2.23, 2.52, and 2.11 times more in *C. epithymum* than in *C. campestris*, respectively. This explained the clustering observed relative to the compounds, where quercetin and dicaffeoylquinic acid were grouped together, while kaempferol-3,7-O-diglucoside and kaempferol 3-O-β-(6′′-O-trans-p-coumaroyl)-glucopyranoside were distant from them (Figure 4). Hyperoside and isoquercitrin had a similar content profile in the samples, forming a group, with chlorogenic acid and kaempferol-3-O-galactoside grouping closest to them in terms of amount in the samples.

## 3. Discussion

The recovery of polyphenolics in alcoholic extracts from both species differed depending on the solvent (Table 2). Due to different polarities, the extraction of various polyphenolics may vary depending on the solvent [26,27], which affected the results for TPC. These results also suggest a higher proportion of highly polar compounds in *C. campestris*, compared with *C. epithymum*. Pure methanol was chosen as a compromise solvent for both species. This may have also affected the number of annotated flavonoids (Figure 2, Table 4), which was lower than usually reported for *Cuscuta* spp. [8,10]. However, except for quercetin of the moderately polar compounds, which was more abundant in *C. epithymum*, this species was also abundant in the highly polar flavonoic glycosides, compared with *C. campestris*, suggesting that the choice of solvent did not significantly affect the obtained flavonoid profiles. Overall, the TPC varied substantially, not only within different samples from the same species, but also within the same sample (Table 3). The slightly higher TPC in *C. epithymum* was also accompanied with higher deviation. All these results suggest that polyphenolic content depends strongly, not only on the environmental conditions in the particular locality [28,29], but also on the host range [14], which is an intrinsic feature of parasitic plants. This would be particularly true for *Cuscuta* spp., considering they are generalists in comparison with the more specialized root parasites of the Orobanchacea family [30,31].

Based on the results obtained from the bioinformatic analysis (Figure 3 and Figure 4), a comparison of flavonoid content between *C. campestris* and *C. epithymum* can be made. The latter contained higher amounts of quinic acid derivatives as well as quercetin and kaempferol derivatives, whereas one of the kaempferol glycosides, kaempferol-3,7-O-diglucoside, as well as kaempferol 3-O-β-(6′′-O-trans-p-coumaroyl)-glucopyranoside were not present in the samples of this species. In a study investigating the phenolic content among several *Cuscuta* species, including *C. campestris*, a division into three groups, based on flavonoid profile, was observed. *Cuscuta campestris* was part of the group of species rich in flavonoid compounds that were characterized by low levels of caffeic acid derivatives [1], which supports the results obtained in the present study. *Cuscuta epithymum* resembled the group of *C. europea* [1], since, in this group, the presence of both caffeic acid derivatives and flavonoid compounds was observed. Differences in flavonoid profile have also been shown for *C. chinensis* and *C. australis*, where the former contained more quinic acid derivative compounds but fewer flavonoids, while the opposite was typical for the latter [10]. This significant difference may be also explained by the phylogenetic distance between the two species, with *C. campestris* belonging to subgenus *Grammica*, and *C. epithymum* to subgenus *Cuscuta* [32].

The higher TPC in *C. epithymum* may be due to the higher amounts of hydroxycinnamic acid derivatives and some flavonoids detected in these samples. Among the samples with the highest levels of total phenolics (Table 3) were C4, C10, and C21 (*C. campestris*) and C6, C9, C13, C15, and C16 (*C. epithymum*), which also featured high levels of annotated flavonoids relative to other samples of the species (Figure 4). For example, C4, C10, and C21 were among the most abundant from *C. campestris* in kaempferol-3,7-O-diglucoside, chlorogenic acid, kaempferol-3-O-galactoside, hyperoside, isoquercitrin, and quercetin. Similarly, the highest levels of flavonoids in *C. epithymum*, including chlorogenic acid, kaempferol-3-O-galactoside, hyperoside, isoquercitrin, dicaffeoylquinic acid, and quercetin, were observed among C6, C9, C13, C15, and C16. A correlation was observed between total phenolic content and levels of flavonoid compounds, confirming flavonoids as a significant fraction of total phenolic content. High levels of total phenol and flavonoid compounds in *C. reflexa* grown on *Coccinia grandis* have been shown to correspond to strong biological activity [12]. Therefore, the study of biological activities of different plants of *C. epithymum* and *C. campestris* would be useful to confirm the relationship between TPC, flavonoid composition, and biological activity.

Dozens of flavonoid compounds have been annotated in *Cuscuta*, the most abundant among the different species being quercetin, kaempferol, and some of their glycosides including hyperoside, quercetin-3-O-glucoside (isoquercitrin), and kaempferol-3-glucoside [8]. In HPLC analyses of the studied *C. campestris* and *C. epithymum* plants collected from different habitats in Bulgaria, the contents of four of the five most abundant flavonoid compounds among *Cuscuta* representatives were detected. Kaempferol-3-glucoside was not isolated in the experiments, but its isomer, kaempferol-3-galactoside, was annotated in the samples. In addition, the presence of kaempferol-3-glucoside in the plants studied was not excluded, as not all compounds present in the plants were annotated due to the focus being primarily on the major flavonoids contained.

## 4. Materials and Methods

### 4.1. Plant Material

A total of 24 samples, 12 representing *C. campestris* and 12 *C. epithymum* were analyzed (Table 1). All samples consisted of vegetative material (stems) from the laboratory collection, collected in the period between 2017 and 2022 between June and August, and stored at −80 °C.

### 4.2. Extraction and Polyphenol Determination

Extraction was initially carried out with pure ethanol and methanol, or 40% and 70% aqueous solutions (*v*/*v*), to choose the optimal solvent. Equal weights of plant tissue—100 mg—were ground in liquid nitrogen and extracted with the respective solvent (HPLC grade) for 12 h under agitation and centrifuged at 12,000× *g* for 10 min at 4 °C. The supernatant was taken up and further analyzed. Total polyphenolic contents (TPCs) were measured by the Folin−Ciocalteu method [33]. Twenty microliters of the plant extract were mixed with 1.58 mL dH_2_O, 0.1 mL of the Folin reagent, and 0.3 mL of 1.8 M Na_2_CO_3_. Following incubation at 40 °C for 30 min in a heat block, absorbance at 765 nm was measured on a UV−Vis spectrophotometer (Jenway 6305, Cole-Parmer Ltd., Stone, Staffordshire, UK), and polyphenol concentrations were calculated as gallic acid equivalents using the molar absorption coefficient of 1.075. Samples were measured in triplicate, where each repetition represented plant tissue, taken from a different part of the stem of the single specimen, taken from the locality. Results were expressed as milligrams of gallic acid equivalents per gram of fresh weight. Statistical significance was determined by one-way ANOVA with Tukey’s post hoc test in GraphPad Prism ver. 8.0.1.

### 4.3. HPLC-MS Analysis

Supernatants were filtered through a Chromafil^®^ PTFE 0.45 µm syringe filter (Macherey-Nagel GmbH & Co. KG, Düren, Germany) and applied into vials. HPLC analyses were performed on the Agilent Technologies 1260 Infinity II LC system (Agilent Technologies, Inc., Santa Clara, CA, USA) including a quaternary pump, autosampler, multicolumn thermostat, WR Diode Array Detector (DAD), and a Quadrupole Time-Of-Flight (QTOF) Agilent 6546 detector. ESI-MS spectra were recorded in negative ion ([M-H]^−^) mode in the interval between *m*/*z* 50 and 1500 and 120 V voltage of the fragmentor. In MS/MS mode, the collision energies were 10 and 20 V. Compounds were separated on a Knauer Eurospher II 100-2 C18 column, 150 × 2 mm, 2 μm particle size. The temperature of the column was 28 °C. Mobile phase consisted of 0.1% formic acid in water (A) and 0.1% formic acid in acetonitrile (B). The gradient at 0.2 mL min^−1^ flow rate was 0–40 min from 0 to 46% B; 40–42 min 100% B; 42–52 min 100% B isocratic; and 52–54 min to 0% B. UV spectra were measured at 190–500 nm.

The Agilent MassHunter Qualitative Analysis, ver. 10.0 was used to visualize chromatograms and peak area integration. Individual flavonoid compounds were annotated based on comparison of the experimental results with literature data with respect to (1) the absorption profile of the molecule; (2) the exact mass of the pseudomolecular ion, found in METLIN and PubChem libraries; (3) the profile of molecular ion fragmentation on MS/MS analysis; and (4) the retention time of the molecular ion. The order of appearance of isomers as well as position of glycosylation were deduced by comparing literature data for characterization of *Cuscuta* extracts from previous studies [10,34]. Mass Profiler Professional 15.1 (Agilent Technologies) was used for statistical analyses through t-test and principal component analysis (PCA) and hierarchic clustering to define flavonoid compounds with significant (*p* < 0.05) differences between *C. campestris* and *C. epithymum*.

## 5. Conclusions

In conclusion, the number of polyphenolic compounds differed significantly between the two *Cuscuta* species studied, between their different populations, and within the same locality, with *C. epithymum* having a slightly higher number. Although the impact of environmental factors and host range may be significant in terms of differences in both polyphenolic concentrations and flavonoid profile, the samples from the two species clearly diverged and clustered separately, suggesting that the flavonoid profile can be used as a suitable chemotaxonomic marker to distinguish them. This is especially useful within the genus *Cuscuta*, where taxonomic identification is often hampered when flowers are missing. These results further emphasized the possibility of extracting bioactive phytochemicals from members of the genus *Cuscuta*, by identifying the most suitable species, as well as by manipulating the quality and quantity of such phytochemicals through the range of host species. This could be of special interest in the countryside, where the collection of medicinal plants has a long tradition, but dodders are often neglected.

## Figures and Tables

**Table 1:** Localities, GPS coordinates, and main host species of *Cuscuta* samples used in the experiments.

*Cuscuta campestris*				*Cuscuta epithymum*			
Sample №	Locality	GPS Coordinates, N, E	Main Parasitized Species	Sample №	Locality	GPS Coordinates, N, E	Main Parasitized Species
C1	St. Nicholas, Chernomorets	42.44795, 27.64192	*Xanthium italicum* Moretti.	C3	Oreshak	42.88511, 24.76739	Not determined
C2	Asenovgrad	42.01476, 24.87629	*Polygonum aviculare* L.	C6	Potochnitsa	41.61129, 25.68116	*Genista rumelica* Velen.
C4	Lozenets	42.21749, 27.78829	*Peucedanum obtusifolium* Sm.	C9	Dragoman	42.94693, 22.93126	*Teucrium chamaedrys* L.
C5	Potochnitsa	41.61120, 25.68446	*Paliurus spina-christi* Mill.	C13	Novo Leski	41.52947, 23.77320	*Teucrium chamaedrys*
C7	Karlanovo	41.54416, 23.41674	*Polygonum aviculare*	C15	Smochevo	42.13189, 23.10084	*Chondrilla juncea*
C8	Mesta	41.79720, 23.66108	*Cichorium intybus* L.	C16	Seslavtsi	42.77921, 23.52794	*Astragalus onobrychis* L.
C10	Disevitsa	43.42416, 24.50320	*Chondrilla juncaea* L.	C17	Smochevo	42.13825, 23.07065	*Sanguisorba officinalis* L.
C11	Dobrinishte	41.82174, 23.56970	*Plantago lanceolata* L.	C18	Smochevo	42.14144, 23.06889	*Nigella damascena* L.
C12	Novo Leski	41.53070, 23.77537	*Daucus carota* subsp. *Sativus* L.	C19	Smochevo	42.14144, 23.06889	*Pinus sylvestris* L.
C14	Smochevo	42.13189, 23.10084	*Chondrilla juncaea*	C22	Mesta	41.75817, 23.67263	*Potentilla argentea* L.
C20	Bebresh	43.00671, 23.82172	*Xanthium italicum*	C23	Mesta	41.75817, 23.67263	*Astragalus onobrychis*
C21	Nova Lovcha	41.42828, 23.73052	*Artemisia campestris* L.	C24	Dragoman	42.94693, 22.93126	*Artemisia alba* Asso.

**Table 2:** Recovery of total polyphenolics in gallic acid equivalents (mg g^−1^ FW) from *Cuscuta campestris* and *Cuscuta epithymum* as dependent on the solvent. Data are mean value ± SD (n = 3). Different letters indicate significant differences at *p* ≤ 0.01 (one-way ANOVA).

Solvent	*Cuscuta campestris*	*Cuscuta epithymum*
40% aq. MetOH	6.30 ± 1.4 ^a^	4.40 ± 0.1 ^b^
40% aq. EtOH	7.85 ± 0.3 ^a^	4.19 ± 0.6 ^b^
70% aq. MetOH	5.06 ± 0.1 ^b^	6.81 ± 1.0 ^a^
70% aq. EtOH	4.00 ± 0.2 ^b^	5.87 ± 0.7 ^ab^
100% MetOH	7.79 ± 1.0 ^a^	8.37 ± 0.8 ^a^
100% EtOH	3.05 ± 0.3 ^b^	9.45 ± 1.5 ^a^

**Table 3:** Total polyphenolic content in gallic acid equivalents (mg g^−1^ FW) in *Cuscuta campestris* and *Cuscuta epithymum* (sample designations correspond to Table 1). Data are mean value ± SD (n = 3). Different letters indicate significant differences at *p* ≤ 0.01 (one-way ANOVA).

*Cuscuta campestris*		*Cuscuta epithymum*	
Sample №	TPC, mg g^−1^ FW	Sample №	TPC, mg g^−1^ FW
C1	3.91 ± 0.4 ^a^	C3	9.64 ± 0.5 ^b^
C2	4.93 ± 0.7 ^a^	C6	11.79 ± 0.5 ^b^
C4	9.83 ± 0.7 ^b^	C9	10.89 ± 0.8 ^b^
C5	6.32 ± 0.6 ^ab^	C13	10.24 ± 0.7 ^b^
C7	4.58 ± 0.3 ^a^	C15	9.33 ± 0.5 ^b^
C8	4.07 ± 0.2 ^a^	C16	10.61 ± 1.0 ^b^
C10	9.35 ± 1.0 ^b^	C17	7.33 ± 0.5 ^ab^
C11	5.10 ± 0.3 ^a^	C18	7.12 ± 0.6 ^ab^
C12	4.78 ± 0.4 ^a^	C19	5.88 ± 0.2 ^a^
C14	6.00 ± 0.4 ^a^	C22	5.89 ± 0.3 ^a^
C20	5.50 ± 0.7 ^a^	C23	4.53 ± 0.6 ^a^
C21	10.51 ± 0.2 ^b^	C24	8.04 ± 0.5 ^b^

**Table 4:** Flavonoid compounds, annotated in *C. campestris* and *C. epithymum* using HPLC-DAD-MS separation/detection on Agilent Technologies 1260 Infinity III LC system. Compound designation corresponds to Figure 2.

Peak	t_R_ (min)	Annotated Compounds	Trivial Name	UV λ Max (nm)	[M-H]^−^ *m*/*z*	MS^2^
1	18.5	Chlorogenic acid		218, 300, 325	353.0858	191.0562, 161.0242
2	21.56	Kaempferol-3,7-O-diglucoside		265, 345	609.1439	447.0890, 285.0392, 489.1018, 327.0496
3	26.572	Quercetin-3-O-galactoside	Hyperoside	255, 355	463.0853	300.0268, 271.0240, 178.9980
4	26.94	Quercetin-3-O-glucoside	Isoquercitrin	255, 355	463.0852	300.0268, 271.02839, 178.9980
5	28.16	Dicaffeoylquinic acid		300sh, 327	515.116	353.0868, 191.0556, 135.0449
6	28.303	Kaempferol-3-O-galactoside		265, 343	447.0902	284.0318, 255.0291, 227.0343, 327.0499, 151.0032
7	29.079	Kaempferol 3-O-glucoside	Astragalin	265, 350	447.0902	284.0318, 255.0290, 227.0343, 327.0500, 151.0032
8	29.146	Isorhamnetin-7-glucoside		255, 353	477.1005	314.0422, 315.0480, 271.0237, 243.0291, 285.0397
9	29.58	Isorhamnetin-3-O-glucoside		255, 345	477.1006	314.0422, 315.0476, 271.0240, 243.0292, 285.0395
10	34.644	Quercetin		255, 370	301.0336	151.0034, 178.9980, 273.0397
11	37.268	Kaempferol 3-O-β-(6′′-O-trans-p-coumaroyl)-glucopyranoside	Tiliroside	268, 315	593.1281	284.0311, 447.0902
12	38.872	Kaempferol		265, 366	285.0389	151.0033, 229.0501, 257.0448, 185.0603
13	39.657	Isorhamnetin		256, 368	315.0492	300.0267, 151.0032, 164.0112, 271.0241, 107.0135

## Data Availability

The original contributions presented in this study are included in the article. Further inquiries can be directed to the corresponding author(s).

## Abbreviations

The following abbreviations are used in this manuscript: DAD—Diode Array Detector; HPLC—high performance liquid chromatography; MS—mass spectrometry; TPC—total polyphenolic content.
